# Daytime QT by Routine 12-Lead ECG Is Prolonged in Patients with Severe Obstructive Sleep Apnea

**DOI:** 10.1155/2020/3029836

**Published:** 2020-02-05

**Authors:** McCall Walker, Jacob N. Blackwell, Patrick Stafford, Paras Patel, Sula Mazimba, Nishaki Mehta, Yeilim Cho, Michael Mangrum, Saman Nazarian, Kenneth Bilchick, Younghoon Kwon

**Affiliations:** ^1^Division of Cardiology, Department of Medicine, University of Texas Southwestern University, USA; ^2^Department of Internal Medicine, University of Virginia, USA; ^3^Division of Cardiovascular Medicine, Department of Medicine, University of Virginia, USA; ^4^Division of Cardiovascular Medicine, Department of Medicine, University of Pennsynlavia, USA

## Abstract

**Background:**

Obstructive sleep apnea (OSA) has been linked to sudden cardiac death (SCD). Prolonged QT is a recognized electrocardiographic (ECG) marker of abnormal ventricular repolarization linked to increased risk of SCD. We hypothesized that individuals with OSA have more pronounced abnormality in daytime QT interval.

**Methods:**

We reviewed consecutive patients who underwent clinically indicated polysomnography with 12-lead ECG within 1 year at a single center. Heart rate-corrected QT interval (QTc) was compared by OSA severity class (normal/mild: apnea‐hypopnea index (AHI) < 15/hr (*n* = 72); moderate: 15-30 (*n* = 72); moderate: 15-30 (*n* = 72); moderate: 15-30 (

**Results:**

A total of 249 patients were included. QTc was similar between the normal/mild and moderate groups, and the overall QTc trend increased across OSA (normal/mild: 435.6 ms; moderate: 431.36; severe: 444.4; *p* trend = 0.03). Abnormal QTc was found amongst 34% of male and 31% of female patients. Patients with severe OSA had longer QTc compared with normal/mild OSA (mean difference (95% CI): 10.0 ms (0.5, 19.0), *p* trend = 0.03). Abnormal QTc was found amongst 34% of male and 31% of female patients. Patients with severe OSA had longer QTc compared with normal/mild OSA (mean difference (95% CI): 10.0 ms (0.5, 19.0), *p* trend = 0.03). Abnormal QTc was found amongst 34% of male and 31% of female patients. Patients with severe OSA had longer QTc compared with normal/mild OSA (mean difference (95% CI): 10.0 ms (0.5, 19.0), *p* trend = 0.03). Abnormal QTc was found amongst 34% of male and 31% of female patients. Patients with severe OSA had longer QTc compared with normal/mild OSA (mean difference (95% CI): 10.0 ms (0.5, 19.0),

**Conclusions:**

In a sleep clinic cohort, severe OSA was associated with higher QTc and clinically defined abnormal QTc compared with nonsevere OSA.

## 1. Introduction

Obstructive sleep apnea (OSA) is a common sleep disorder affecting approximately 3 to 7% of adult men and 2 to 5% of adult women worldwide [1], with estimates of greater than 20 million people affected in the United States alone [2]. OSA has been associated with increased cardiovascular risks including hypertension, coronary artery disease, heart failure (HF), and atrial fibrillation (AF) [3–5]. Recently, OSA has also been linked to sudden cardiac death (SCD) [6]. In patients with HF, both OSA and central sleep apnea are associated with higher rates of appropriate defibrillation by implantable cardiac defibrillator, a surrogate for malignant ventricular arrhythmia [7]. Although the mechanism is unclear, OSA's association with SCD may be partly mediated by change in the underlying resting ventricular repolarization in patients with OSA [8].

The QT interval represents a period between the onset of ventricular depolarization and the end of the repolarization. Through changes in cardiac ion channels (namely, blockade of the “rapid” and “slow” potassium currents), the cardiac action potential becomes prolonged which can create a proarrhythmic effect manifested on electrocardiogram (ECG) as a prolonged QT interval [9]. This can provoke ventricular arrhythmias, most notably torsades de pointes. Prolonged QTc and QTc interval dispersion (the difference between the longest and shortest QTc measured on ECG) have been shown to be independent risk factors for SCD [10, 11].

The QT interval has been previously examined in the setting of OSA in limited capacity mostly with use of overnight Holter monitoring [12] and with single-lead ECG [8]. Other studies have focused on QT dispersion in OSA however only in young children [13]. ECG is a readily available tool, and thus, prolonged QT has a potential as a useful tool to better risk stratify patients with OSA for adverse outcomes such as SCD. The main purpose of this study was to compare QT across OSA severity groups in patients with high baseline cardiovascular risks.

## 2. Materials and Methods

### 2.1. Subjects

Consecutive patients who were referred to the single academic sleep center for diagnostic polysomnography (PSG) for high suspicion of OSA from January 1, 2010, through December 31, 2016, were included in the study. Inclusion criteria were as follows: (1) age ≥ 20 years, (2) normal sinus rhythm on 12-lead surface ECG, and (3) ECG obtained within 1 year following PSG. If multiple ECGs were available, the one closest to the date of PSG was selected. Subjects with a documented history of QTc-prolonging medications (including but not limited to certain antibiotics, antipsychotics, antiarrhythmics, or beta-blockers) or bundle branch block were excluded from the initial analysis. Patients with a history of AF were included, but no patient demonstrated a rhythm of AF in the ECG chosen for this study. Demographic information (age, sex, and race), body mass index (BMI), and medical history (hypertension, chronic obstructive pulmonary disease, AF, and HF) were obtained from electronic medical records. Clinical history of each patient was meticulously chart reviewed on an individual basis for discrepancies in diagnoses as well as verification of additional patient information. HF was defined as including those patients with depressed ejection fraction or those with preserved ejection fraction but with clinical symptoms of HF. This study was approved by the institutional review board at the University of Virginia.

### 2.2. Sleep Study Data

Overnight PSG was performed employing the standard recommendation of the American Academy of Sleep Medicine (AASM), and data were processed with Embla Sandman Elite software (Natus Medical Incorporated, California, USA). For this study, only those subjects who underwent full-night diagnostic PSG were included. The apnea-hypopnea index (AHI) was defined as the number of apnea and hypopnea events divided by the total sleep time and expressed as the number of events per hour. Apnea was defined as a reduction in airflow greater than 90% of the preevent baseline, occurring for longer than 10 seconds using a thermocouple signal. Hypopnea events were recorded when the amplitude of the nasal pressure flow signal decreased by more than 30% of the preevent baseline for longer than 10 seconds; only hypopneas with 4% desaturation were included in the AHI reported here. Severity of OSA was classified as normal/mild: AHI < 15/hr (reference), moderate: AHI 15-30, and severe: AHI > 30. Mean oxygen (O_2_) saturation was also utilized as an alternative measure of OSA severity.

### 2.3. Electrocardiography

Standard 12-lead ECGs (Philips PageWriter TC70; GE, Eindhoven, Netherlands) were digitally recorded at a speed of 25 mm/sec with a calibration of 10 mm/mV at resting conditions (seated for at least 5 minutes with the arm at heart level) during daytime clinic hours. All ECGs were assessed visually for inadequate quality, processed digitally via an ECG software platform (TraceMaster-Philips IntelliSpace ECG; Philips Medical Systems, Andover, MA, USA), and confirmed visually by physicians. The same software program was used to automatically measure QT and calculate QTc. QTc was calculated using the Bazzett formula. Investigators extracting ECG measures were blinded to clinical and PSG data.

### 2.4. Statistical Analysis

Comparisons were made across the OSA severity groups using chi-square test for categorical variables and ANOVA for continuous variables. We constructed a multiple regression model adjusting for age, sex, BMI, HF, and hypertension status to determine the difference in mean QTc across the OSA severity groups. Further analysis additionally adjusting for race, AF, and COPD did not change the results.

Using the same covariates, we performed multivariable logistic regression to determine the association between AHI class and abnormal QTc (duration defined as >450 and >470 ms for male and female, respectively).

Based on no significant difference in QTc between the normal-mild and moderate OSA groups, we grouped patients into dichotomous groups as nonsevere vs. severe OSA and repeated the multivariate analyses. Given the significant interaction by HF status (*p* = 0.07), we also performed the analyses stratified by HF status. As a part of sensitivity analyses, we again repeated analyses after including patients with bundle branch block (*N* = 26) and additionally adjusting for QRS duration.

The sample size of each group (*N* = 105 for severe, *N* = 144 for nonsevere), mean QTc values of 444.4 ms (severe) and 433.5 ms (nonsevere), and SD of 30 gave us power (1-beta) of 0.81 at an alpha of 5%.

All outcome results were expressed per 1 standard deviation of predictor values. *p* values less than 0.05 were considered significant. All analyses were performed using Statistical Analysis System (SAS) software version 9.0 (Cary, NC).

## 3. Results

**Figure 1:**
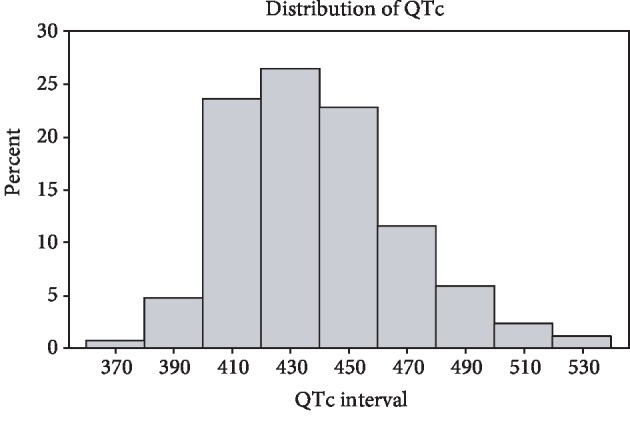
Distribution of QTc amongst total study population by percentage.

Demographic and clinical data for the study population are detailed in Table 1. QTc distribution amongst the total study population is shown in Figure 1. A total of 249 patients consisting of 49% men were included. The mean age (SD) of the cohort was 57 (12) years old, 50.2% were female, and 22% were nonwhite. The majority of patients had OSA (normal/mild: *n* = 72; moderate: *n* = 72; severe: *n* = 105). Patients with greater AHIs tended to be men and to be more obese. In all patients, the mean AHI was 35.3/hr (SD 29.7) and the mean O_2_ saturation was 92.4% (SD 3.0). An abnormal QTc was found amongst 34% and 31% of the male and female patients, respectively.

Overall, QTc was similar between the normal/mild and moderate groups and the overall QTc trend increased across OSA (normal-mild: 435.6 ms; moderate: 431.36 ms; severe: 444.4 ms; *p* for linear trend 0.03) (Table 2). In multivariable analysis, patients with severe OSA had higher QTc compared to those with normal-mild OSA (mean difference (95% CI): 10.0 ms (0.5, 19.0), *p* = 0.04). Adjusted mean QTc values for each AHI class based on multiple regression are shown in Table 2. When examining OSA's association with clinically defined abnormal QTc as an outcome, no association was found between either moderate or severe OSA and abnormal QTc (OR (95% CI): 0.78 (0.36, 1.67), *p* = 0.1 (moderate vs. ref); 1.44 (0.72-2.92), *p* = 0.19 (severe vs. ref)).  Table 3 shows factors including covariates that are associated with abnormal QTc. The results remained similar when patients with bundle branch block were included and when analysis additionally adjusted for race, AF, and COPD was included (data not shown).

**Figure 2:**
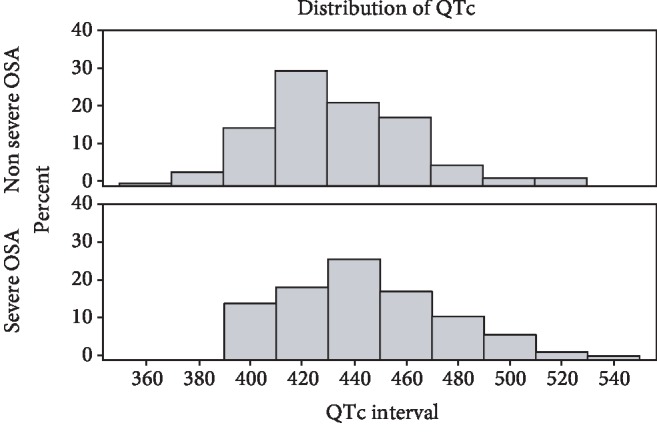
Distribution of QTc amongst patients with severe OSA vs. nonsevere OSA by percentage. OSA = obstructive sleep apnea.

**Figure 3:**
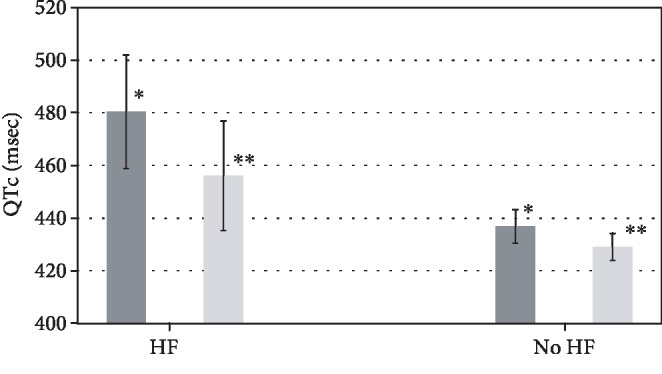
QTc based on severity of OSA. Adjusted mean QTc values of subsequent analysis (severe OSA vs. nonsevere OSA). *p* value = 0.03 for severe vs. nonsevere OSA in patients with HF; *p* value = 0.06 for severe vs. nonsevere OSA in patients without HF. OSA = obstructive sleep apnea; HF = heart failure. ^∗^Patients with severe OSA. ^∗∗^Patients without severe OSA.

In a subsequent analysis stratifying patients based on severe vs. nonsevere OSA (AHI ≥ 30/hr vs. <30, where nonsevere includes normal-mild and moderate OSA), severe OSA was associated with a significantly longer QTc than nonsevere (Table 4). QTc distribution amongst patients stratified by severe vs. nonsevere OSA is shown in Figure 2. Moreover, severe OSA (vs. nonsevere OSA) was associated with a clinically abnormal QTc (Table 3). HF status-stratified analysis showed that the difference in QTc by OSA status (severe vs. nonsevere) was more prominent in patients with HF (QTc, adjusted, patients with HF 480.47 ms (458.85-502.08) for severe OSA, 456.05 ms (435.28-476.81) for nonsevere OSA; QTc, adjusted, patients without heart failure 436.87 ms (430.49-443.25) for severe OSA, 429.09 ms (423.93-434.25) for nonsevere OSA) (Figure 3). Similarly, odds ratio of abnormal QTc was more prominent in those with HF (Table 3).

## 4. Discussion

In a sleep clinic cohort with a high burden of cardiovascular risk, we found that patients with severe OSA had prolonged daytime QTc compared to patients with nonsignificant OSA (normal to mild OSA). While there was no significant difference in QTc between patients with normal/mild and moderate OSA based on AHI, there was a significant difference when comparing either of these groups to the severe OSA group. Analysis of our initial stratification prompted further inquiry into driving factors of clinically abnormal QTc given the degree that the severe OSA group distinguished itself from the other OSA severity groups. After patients were dichotomized into severe vs. nonsevere OSA, a clinically abnormal QTc became evident in the severe group. This may suggest that patients with severe OSA represent a distinct group from those with normal-mild or moderate (collectively nonsevere) OSA in terms of its association with QTc.

OSA is a highly common sleep disorder that is increasingly recognized as an independent risk for CVD [14]. A limited but emerging body of research has further elaborated on the link between OSA and SCD. A clinic-based cohort study by Gami et al. showed that the lowest nocturnal O_2_ saturation at night, one of the markers of OSA severity, was independently predictive of SCD [6]. In addition, people with OSA were associated with higher predilection of nocturnal SCD compared to others without documented history of OSA [15]. Although the mechanism of such an association would be likely multifactorial, a number of studies have suggested electrocardiographic changes as one mechanism or as a marker to explain the relationship. Çiçek et al. showed an increase in average QTc from 24-hour Holter monitor recording in newly diagnosed, untreated patients with OSA [12]. Similarly, Shamsuzzaman et al. demonstrated that OSA is associated with a significant increase in resting daytime QTc based on continuous 10-minute supine resting ECG [8]. OSA has also been associated with a higher prevalence of other ECG findings representing high-risk features for ventricular arrhythmia such as QT dispersion, Tp-e interval, Tp-e/QT ratio, and QRS-T angle [16–18].

Through mediation of sympathetic/parasympathetic balance (and therefore changes in vagal tone), QT interval tends to increase in sleep as compared to the awake state in the general population [19–21]. Patients with OSA have a further increase in nocturnal QTc mediated by intermittent hypoxia which in turn can cause severe sympathetic activation during both night and daytime [22–24]. Thus, prolongation of daytime baseline QT interval in patients with severe OSA as shown in our study may be mediated by exposure to chronic hypoxia and is one mechanism that explains higher risk of SCD in patients with OSA [6, 7], and exaggerated dynamic change in QT may be responsible for SCD events in patients with OSA [15, 22].

Our cohort was characterized by significant comorbidities that place them at a much higher cardiac risk than the general population and patient cohorts included in other similar studies [8]. Compared to the general population, our cohort had significantly higher percentage of those with prolonged QTc (34% in male and 31% in female in this study, 8.7% of the general population) [25]. Considering the high prevalence of sleep-disordered breathing including OSA in patients with HF, our cohort represents a high cardiovascular risk group wherein QT interval may have more meaningful implications than in the general population. Specifically, twenty percent of our patients had a diagnosis of HF in addition to OSA, making this a particularly vulnerable group of patients. As demonstrated in our study as well as other studies, HF is a potent risk for prolonged QTc [26]. The more prominent association of severe OSA with QTc in patients with HF shown in our study suggests that there may be synergistic effect between OSA and HF in their joint effects on QTc.

The clinical implications of our findings are far-reaching. QT prolongation in the setting of OSA seems to be relevant particularly in severe OSA (as opposed to nonsevere), and this should be taken into consideration in all patients when evaluating risk of morbidity including SCD and particularly in those patients at a higher baseline risk of SCD, such as patients with coexisting HF. Our findings suggest that earlier screening for OSA in patients with concomitant cardiac disease may be warranted to potentially impact morbidity and mortality, specifically from the standpoint of SCD. This being said, it is still uncertain whether treatment can truly impact or reverse risk of SCD, although interpretation of recent data suggests that QTc and ventricular ectopy can be reduced with treatment of OSA [27, 28].

A major strength of our study includes meticulous exclusion of patients on any medication that may impact QTc. Particular attention was paid to account for other potential influencing factors on QT interval including age, sex, BMI, HF, and hypertension. Furthermore, given increasing complexity of patients being referred to tertiary sleep laboratories undergoing in-lab PSG, inclusion of many patients with high cardiovascular risk profiles in our study represents a cohort that is more likely to be seen in daily clinical practice than in previously conducted studies.

Several weaknesses of the study are worthy of note. ECGs that were assessed were not necessarily recorded the same day as the PSG was completed and in the majority of cases were recorded at least weeks after PSG. While not ideal, these limitations are not critical and likely have little effect on the overall significance given the chronic nature of OSA. We assume that many patients with severe OSA and to a lesser extent nonsevere OSA may have been treated with continuous positive airway pressure at the time of ECG which has the chance of biasing our results. However, in the case that continuous positive airway pressure improves QT interval more in severe OSA as opposed to nonsevere OSA (although not clear based on current evidence), our results actually may have been more striking if those who received treatment were excluded. Our study is inherently subject to the bias of a retrospective approach. Despite this, findings of our study propose an important question as to the possible role of QT interval in further risk assessment of patients with severe OSA. Future studies should consider linking prolonged daytime QTc in patients with OSA with clinically meaningful outcomes such as SCD.

## 5. Conclusion

We found that severe OSA is associated with an increase in daytime QTc, a marker of ventricular repolarization.

## Figures and Tables

**Table 1:** Baseline characteristics of the study population stratified by category of obstructive sleep apnea (OSA).

Variable	All (% or SD)	Normal-mild (% or SD)	Moderate (% or SD)	Severe (% or SD)	*p* value
	(*N* = 249)	(*N* = 72)	(*N* = 72)	(*N* = 105)	
Age (years)	57.2 (12.5)	56.4 (13.4)	57 (11.8)	57.8 (12.4)	0.74
Sex (female)	125 (50.2%)	49 (68%)	35 (48.6%)	41 (39.1%)	0.0007
Race	White 194 (77.9%)	53 (73.6%)	64 (88.9%)	77 (73.3%)	
	Black 47 (18.8%)	17 (23.6%)	7 (9.7%)	23 (21.9%)	0.1
	Other 8 (3.2%)	2 (2.8%)	1 (1.4%)	5 (4.8%)	
Hypertension	182 (73.1%)	48 (66.7%)	48 (66.7%)	86 (89.9%)	0.03
HF	50 (20.1%)	13 (18.1%)	12 (16.7%)	25 (23.8%)	0.45
COPD	40 (16.1%)	20 (27.8%)	9 (12.5%)	11 (10.5%)	0.005
AF	43 (17.3%)	18 (25%)	7 (9.7%)	18 (17.1%)	0.052
BMI (kg/m^2^)	37.2 (9.4)	34.3 (8.7)	36 (8)	39.9 (10.1)	0.0002
AHI (/hr)	35.3 (29.7)	8.78 (3.79)	21.18 (4.3)	63.21 (25.93)	NA
Mean oxygen saturation (%)	92.7 (3)	93.6 (2.7)	93.2 (2.3)	91.6 (3.3)	<0.0001
On AV blockades	129 (52.2%)	34 (48.6%)	32 (44.4%)	63 (60%)	0.097

Values are means (SD) for continuous variables and percentages for dichotomous variables. Classification of apnea-hypopnea index (AHI): normal-mild: <15/hr, moderate: 15-30/hr, and severe: >30/hr. HF = heart failure; COPD = chronic obstructive pulmonary disease; AF = atrial fibrillation; BMI = body mass index; AV block = atrioventricular block; SD = standard deviation.

**Table 2:** QTc based on OSA severity.

OSA severity	Normal-mild (SD or 95% confidence limits)	Moderate (SD or 95% confidence limits)	Severe (SD or 95% confidence limits)	*p* value
QTc (ms, unadjusted)	435.60 (29.36)	431.36 (27.88)	444.40 (30.86)	0.01^∗^, 0.03^†^
QTc (ms, adjusted)	441.07 (433.26-448.88)	438.78 (431.3-446.26)	450.83 (444.05-457.6)	0.04^∗^, 0.007^‡^, 0.63^¥^

Adjusted and unadjusted mean QTc values. Classification of apnea-hypopnea index (AHI): mild: <15/hr, moderate: 15-30/hr, and severe: >30/hr. OSA = obstructive sleep apnea. Adjusted results are based on multivariable regression adjusting for body mass index, age, gender, presence of hypertension, and presence of heart failure. Analysis additionally adjusting for race, atrial fibrillation, and chronic obstructive pulmonary disease did not change the results (not shown). ^∗^Severe compared to normal-mild OSA. ^‡^Severe compared to moderate OSA. ^¥^Moderate compared to normal-mild OSA. ^†^*p* value for linear trend (unadjusted) across OSA severity classes.

**Table 3:** Odds ratio of abnormal QTc based on comorbidity.

Initial analysis	Odds ratio	95% CI	*p* value
Moderate vs. normal-mild OSA	0.78	0.36-1.67	0.19
Severe vs. normal-mild OSA	1.44	0.72-2.92	0.1
BMI (kg/m^2^)	0.99	0.95-1.02	0.4
Age (years)	0.99	0.97-1.02	0.6
Hypertension	1.36	0.66-2.88	0.41
HF	4.07	2.1-8.06	<0.0001
Subsequent analysis (dichotomous groups)	Odds ratio	95% CI	*p* value
Severe vs. nonsevere OSA	2.68	1.34-5.48	0.0058
BMI (kg/m^2^)	0.96	0.92-1	0.05
Age (years)	0.99	0.96-1.02	0.62
Hypertension	1.42	0.59-3.67	0.45
HF	5.41	2.63-11.35	<0.0001

Classification of apnea-hypopnea index (AHI) in trichotomous stratification: normal-mild: <15/hr, moderate: 15-30/hr, and severe: >30/hr. For dichotomous stratification: nonsevere: <30/hr (i.e., normal-mild and moderate), severe: >30/hr. OSA = obstructive sleep apnea; BMI = body mass index; HF = heart failure; CI = confidence interval.

**Table 4:** QTc based on severity of OSA (severe vs. nonsevere).

Variable	Severe OSA (95% confidence limits)	Nonsevere OSA (95% confidence limits)	*p* value
QTc (ms, unadjusted)	444.4 (438.71-450.09)	433.48 (428.62-438.33)	0.0044^∗^
QTc (ms, adjusted)	450.89 (444.13-457.65)	439.86 (433.85-445.87)	0.0051^∗^

Mean QTc values of subsequent analysis based on dichotomous groups (severe OSA, nonsevere OSA). Nonsevere OSA includes normal-mild and moderate OSA. OSA = obstructive sleep apnea. ^∗^Severe compared to nonsevere OSA.

## Data Availability

Data used in this original manuscript can be found in Excel spreadsheets/datasets personally generated by the authors and can be provided to *Sleep Disorders*/Hindawi upon request.
